# Comparative pharmacokinetic study of the five anti-inflammatory active ingredients of *Inula cappa* in a normal and an LPS-induced inflammatory cell model

**DOI:** 10.3389/fphar.2022.981112

**Published:** 2022-09-19

**Authors:** Jing Huang, Ruixing Chen, Jie Zhou, Qing Zhang, Cun Xue, Yueting Li, Lin Zheng, Yong Huang, Qun Wang, Yi Chen, Zipeng Gong

**Affiliations:** ^1^ State Key Laboratory of Functions and Applications of Medicinal Plants, Guizhou Provincial Key Laboratory of Pharmaceutics, School of Pharmacy, Guizhou Medical University, Guiyang, China; ^2^ Guizhou University of Traditional Chinese Medicine, Guiyang, China; ^3^ Guizhou Provincial Engineering Research Center for the Development and Application of Ethnic Medicine and TCM, Guizhou Medical University, Guiyang, China

**Keywords:** *Inula cappa*, cellular pharmacokinetics, UPLC-MS/MS, RAW264.7 cells, inflammatory model

## Abstract

*Inula cappa* is a commonly used medicine in the Miao area of Guizhou Province in China. We established an *in vitro* inflammatory model of mouse macrophage RAW264.7 cells to study the different pharmacokinetics of five anti-inflammatory active ingredients in the *I. cappa* extract namely luteolin (LUT), chlorogenic acid (CA), cryptochlorogenic acid (CCA), 3,4-dicaffeoylquinic acid (3,4-DCQA) and 4,5-dicaffeoylquinic acid (4,5-DCQA), in a normal and an inflammatory cell model. First, RAW264.7 cells were treated *in vitro* with l μg/mL lipopolysaccharide (LPS) for 24 h to establish an inflammatory cell model. Then, the pharmacokinetic characteristics of the five ingredients were compared in normal and inflammatory cells after treatment with 200 μg/ml and 800 μg/ml of *I. cappa* extracts. After treatment with 1 μg/ml LPS for 24 h, the volume of RAW264.7 cells was increased, the morphology was changed, the antennae were obvious, and the secretion of inflammatory factors nitric oxide and TNF-α was increased. The pharmacokinetics results showed that the five ingredients in normal and inflammatory cells exhibited an increase in C_max_ and AUC values with increasing doses, and the C_max_ and AUC values of five ingredients were positively correlated with the extract concentration. Each of these five ingredients presented nonlinear pharmacokinetic characteristics. After treatment with 200 μg/ml of *I. cappa* extract, the uptake of five ingredients increased in inflammatory cells, T_max_ was prolonged, MRT and t_1/2_ were prolonged, and CL_F and Vz_F were decreased, while after treatment with 800 μg/ml of *I. cappa* extract, the uptake of five ingredients decreased, T_max_ was prolonged, absorption was faster, and MRT and t_1/2_ were prolonged. The five analyzed components in *I. cappa* extract exerted different effects on normal cells and LPS-induced inflammatory cells. Compared to normal cells, the uptake of five ingredients in inflammatory cells was faster and the AUC and C_max_ values increased with increasing doses, showing a dose-dependent nonlinear pharmacokinetic profile. These results indicate that the pharmacokinetic effects of the five analyzed ingredients in *I. cappa* extract are changed in the inflammatory state.

## 1 Introduction

*Inula cappa* (Buch.-Ham. ex D. Don) DC. is a member of the Asteraceae family whose dried whole herb or root is used as a traditional Chinese medicine. It is also called BaiNiudan, DaLiwang, or YeXiabai. *I. cappa* is distributed in Guizhou, Yunnan, Guangxi, and Hunan Province in China. Its properties and functions are described in the Provincial Quality Standards for Medicinal Materials (Guangxi Zhuang Autonomous Region, Department of Health, 1992; Guizhou Province Drug Administration, 2003; Yunnan Food and Drug Administration, 2007; Hunan Food and Drug Administration, 2010). As a commonly used medicine in the Miao region of Guizhou Province, *I. cappa* has the function of dispersing wind-heat, detumescence, and detoxification. It is used for the treatment of cold and fever, swelling and pain of the throat, rheumatism arthralgia pain, ulcerative carbuncle, and furunculosis, among others (Qiu and Du, 2005). Among the Dai people, *I. cappa* also known as “Nahan”, is the main medicine of the famous Dai medical prescription “Ya Hutton San”, which is included in the 2020 edition of the Chinese Pharmacopoeia. *I. cappa* has the effect of clearing away heat and toxic materials, analgesia, and hemostasis, and it is used in the treatment of cold and fever, pharyngitis, thoracoabdominal pain, palpitation, deficiency, irregular menstruation, and postpartum bleeding (Chinese Pharmacopoeia Commission, 2020). *I. cappa* is mainly composed of terpenoids, flavonoids, phenols, organic acids, and volatile oil (Xie, et al., 2007; Yao and Liang, 2008; Wu, et al., 2010; Hu and He, 2012; Guan, et al., 2014; Wu, et al., 2017; Yao, 2017; Zhou, et al., 2017). Modern pharmacological studies have indicated that *I. cappa* has many biological activities and exerts anti-inflammatory, analgesic, antibacterial, antitumor, antioxidant, and immunomodulatory effects (Liu et al., 2009; Liu, et al., 2010; Jiraporn, et al., 2011; Xie, et al., 2012; Dong, et al., 2015; Li, 2017). Compared with the many studies on the pharmacological properties and chemical composition of *I. cappa*, there are few pharmacokinetic studies on its active ingredients, and cellular pharmacokinetic studies on its active ingredients are still lacking. In fact, classical pharmacokinetic studies based on the determination of plasma drug concentrations may not be sufficient to predict pharmacological response *in vivo*. Many drugs must pass through multiple biological barriers before they reach the intracellular target. There is a pressing need to expand classic pharmacokinetics from macroscopic plasma concentrations of drugs to the cellular or subcellular levels. In this context, cellular pharmacokinetics came into being.

Cellular pharmacokinetics is focused on the quantitative study of the kinetic processes of absorption, distribution, metabolism, and excretion of drugs in cells, which could elucidate the disposition pattern of drugs in cells and evaluate the efficacy of drugs by establishing mathematical models (Ni, et al., 2014; Liu, et al., 2021). It is worth noting that studies have shown that at least one-third of drug targets are currently located in cells, including DNA, nuclear receptors, various kinases, and metabolic enzymes. The representative drugs include antibiotics (azithromycin, moxifloxacin), antimalarial drugs (chloroquine), anticancer drugs (doxorubicin, paclitaxel, topotecan) and antidiabetic drugs (berberine). Drugs with these targets located in cells must penetrate multiple biological barriers and bind to intracellular targets to exert their efficacy. The process of intracellular drug disposal and the binding of the drug to the target are the determinants of drug therapy efficacy. For drugs whose targets are located in cells, it may be more important to study the time course of the drug concentration in cells/subcellular organelles than to study the plasma drug concentration. Therefore, from the perspective of cellular pharmacokinetics, this study regards cells as a microscopic organic whole, quantitatively studies the kinetic process of drugs in cells, then clarifies the laws of drug disposal in cells, and scientifically evaluates the drug efficacy.

For some traditional Chinese medicines whose targets are located in cells, the free drug concentration in the target cells is related to pharmacology, that is, the target that produces the drug’s effect may also exist in the cell; so, the intracellular free drug concentration affects the target inside the cell (Mateus, et al., 2013). Therefore, it is particularly important to measure the free concentration of the drug in target cells. Moreover, the analysis of the pharmacokinetic processes of drugs inside cells is closely related to its production efficacy. In inflammatory states, the presence of inflammatory factors affects the kinetic disposition of drugs in the body, which leads to the pharmacokinetic parameters of the drugs in the body being different from that of organisms in the normal state. Therefore, it is more relevant to study the pharmacokinetic characteristics of drugs in the inflammatory state than in the normal state (Gao, et al., 2011).

**FIGURE 1:**
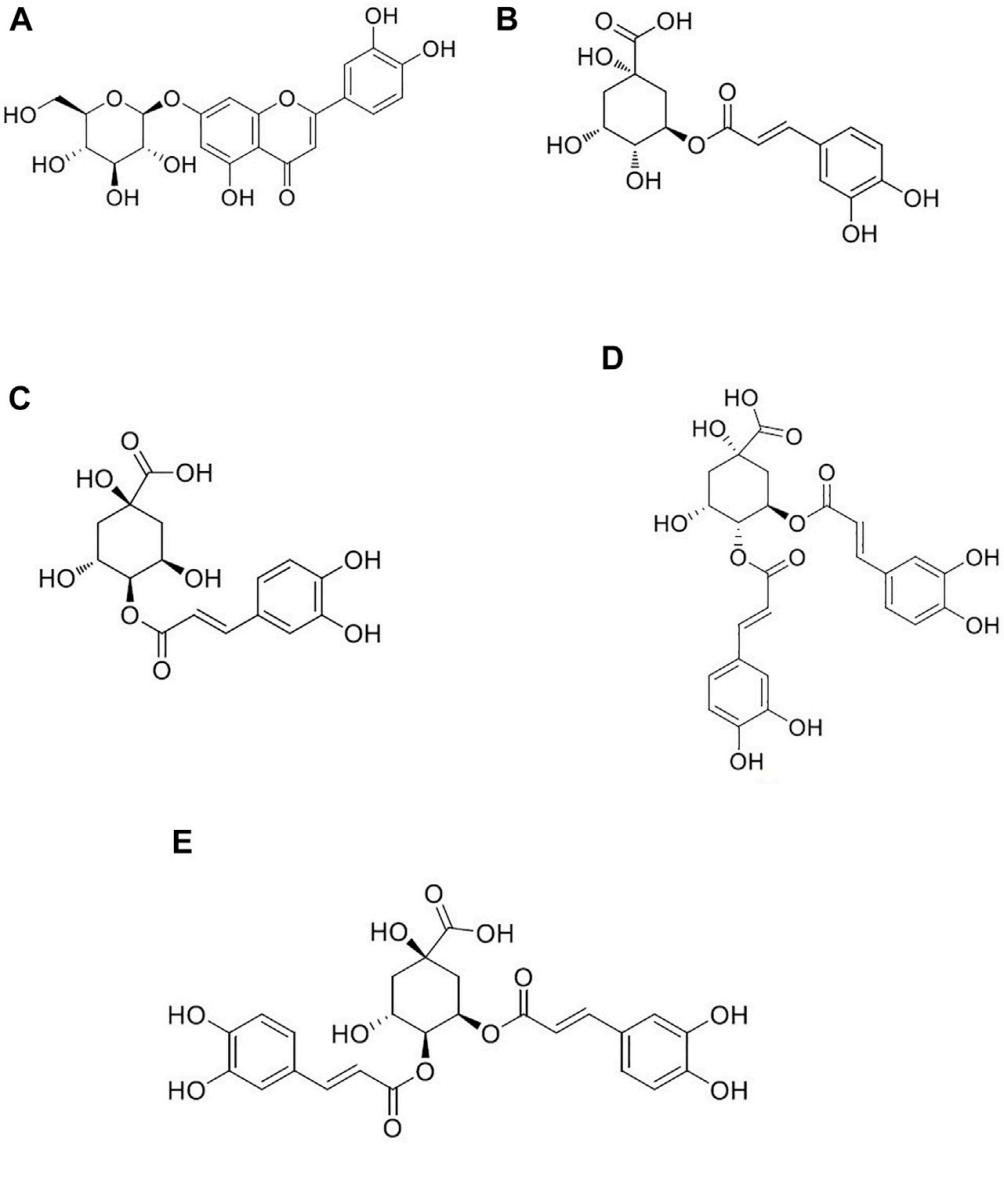
Chemical structures of luteolin **(A)**, chlorogenic acid **(B)**, cryptochlorogenic acid **(C)**, 3,4-dicaffeoylquinic acid **(D)**, and 4,5-dicaffeoylquinic acid **(E)**.

Our previous study completed the fingerprint and chemical composition identification of the extract of *I. cappa* (Gong, et al., 2017b; Zhang, et al., 2019). Moreover, we revealed that the 60% ethanol fraction of *I. cappa* has shown good anti-inflammatory effects *in vitro* and *in vivo* (Gong, et al., 2017a; Wang, et al., 2018). The active ingredients of the 60% ethanol fraction included caffeic acid compounds, phenylpropanoids, and flavonoids, and the main ingredients showing anti-inflammatory activity entering the blood circulation are luteolin (LUT), chlorogenic acid (CA), cryptochlorogenic acid (CCA), 3,4-dicaffeoylquinic acid (3,4-DCQA) and 4,5-dicaffeoylquinic acid (4,5-DCQA) (Gong, et al., 2017c). In addition, our previous study evaluated the absorption and transport properties of active ingredients from *I. cappa* extract using a Caco-2 cell model and showed that the uptake and transport of five active ingredients in Caco-2 cells exhibited a first-order kinetic process (Xue, et al., 2020). Therefore, in the present study, the five ingredients, namely LUT, CA, CCA, 3,4-DCQA, and 4,5-DCQA were used as indicators to study cellular pharmacokinetics of *I. cappa* extracts, and Figure 1 shows the chemical structures of the five ingredients. Firstly, the drug concentrations of the five active components were determined in normal and LPS-induced RAW264.7 cells after treatment with 200 μg/ml and 800 μg/ml of *I. cappa* extracts. Then, the pharmacokinetic parameters were calculated with WinNonLin software to reveal the changes of the five active ingredients over time. Finally, the effects of inflammation on the cellular pharmacokinetics of the active ingredients of *I. cappa* were investigated by comparing the pharmacokinetic parameters of the five active ingredients at different concentrations between the normal and inflammatory states.

## 2 Materials and methods

### 2.1 Instruments

The following instruments were used: UPLC Xevo TQ-S Ultra Performance Liquid Triple Quadrupole Mass Spectrometer (ACQUITY UPLC I-Class System, MassLynx Mass Spectrometry Workstation, Waters Corporation, United States); CO_2_ incubator, ultra-low temperature refrigerator (Thermo Fisher Scientific, United States); Model 680 Enzyme Labeler (Burroughs Life Medical Products Co., Ltd.); TS-100 F inverted microscope (Nikon Corporation, Japan); Ultra Clean Bench; Allegra 32 R Benchtop Refrigerated Centrifuge; Allegra X-30 R low-temperature high-speed centrifuge (Beckman, United States); VX-III multi-tube vortex oscillator (Fujikin (Beijing) Pharmaceutical Technology Co., Ltd.); XYN-15LP nitrogen blowing instrument nitrogen generator (Shanghai Analytical Instruments Co., Ltd.); Water purifier (Sichuan Water Treatment Equipment Co., Ltd.).

### 2.2 Reagents and drugs

The following agents were purchased: puerarin (China Institute of Food and Drug Administration, Lot No. 110752-201514, purity ≥95.5%); CA (Chengdu Efa Biotechnology Co., Ltd., Lot No. AF8112791, purity ≥98%); CCA (Chengdu Efa Biotechnology Co., Ltd., Lot No. AF20032707, purity ≥98%); LUT (Sichuan Vickers Biotechnology Co., Ltd., Lot No. wkq20031803, purity ≥98%); 3,4-DCQA (Chengdu Efa Biotechnology Co., Ltd., Lot No. AF9060515, purity ≥98%); 4,5- DCQA (Chengdu Efa Biotechnology Co., Ltd., Lot No. AF0011701, purity ≥98%).

The mouse mononuclear macrophage RAW264.7 cell line used in this experiment was purchased from Wuhan Puno Life Sciences Co. (No. CL-0910). Australian fetal bovine serum (FBS) (2206991CP), DMEM (8120518), double antibody (15140-122), and Tryptic digest (25200-056) were all purchased from Gibco, United States. LPS (L8880) and the BCA protein quantification kit were purchased from Beijing Solai Bao Technology Co.; the NO one-step kit (A013-2-1) was purchased from Nanjing Jiancheng Institute of Biological Engineering; the TNF-α ELISA kit (KE10002) was purchased from Wuhan Sanying Biotechnology Co.

*I. cappa* was identified by associate Professor Chunhua Liu, School of Pharmacy of Guizhou Medical University. *I. cappa* extract was prepared as described in our previous study. The contents of LUT, CA, CCA, 3,4-DCQA, and 4,5-DCQA in the *I. cappa* extract were 0.18%, 0.26%, 0.30%, 4.52%, and 4.36%, respectively.

### 2.3 Methods

#### 2.3.1 Solution preparation

##### 2.3.1.1 Preparation of internal standard solution

We precisely weighed 5.7 mg of puerarin, placed into a 5 ml brown volumetric flask and added methanol to obtain a stock solution of 2.74 mmol/L, which was prepared to the proper concentration and stored at −20°C.

##### 2.3.1.2 Preparation of control solution

We accurately weighed 5.7 mg of luteolin, 10.13 mg of chlorogenic acid, 10.28 mg of cryptochlorogenic acid, 15.21 mg of 3,4-dicaffeoylquinic acid, and 15.17 mg of 4,5-dicaffeoylquinic acid. The corresponding concentrations of 2.25, 5.67, 5.79, 5.85, and 5.83 mmol/L were obtained by adding methanol to 5 ml of standard stock solution and stored at −20 °C; the stock solution was diluted to mixed standard solutions before use (LUT, CA, CCA, 3, 4-DCQA, and 4, 5-DCQA were 0.14, 0.35, 0.35, 0.95, and 0.96 μmol/L, respectively). Finally, as shown in Table 1, we diluted the standard solutions by multiples to the series concentrations.

**TABLE 1:** The standard series concentration of five ingredients including LUT, CA, CCA, 3,4-DCQA, and 4,5-DCQA (μmol/L).

**Ingredients**	**The number of standard series concentration**							
		**1**	**2**	**3**	**4**	**5**	**6**	**7**	**8**
LUT	0.14	0.069	0.034	0.017	0.0086	0.0043	0.0022	0.0011
CA	0.35	0.18	0.088	0.044	0.022	0.011	0.0055	0.0028
CCA	0.35	0.18	0.088	0.044	0.022	0.011	0.0055	0.0028
3,4-DCQA	0.95	0.48	0.24	0.12	0.059	0.030	0.015	0.0075
4,5- DCQA	0.96	0.48	0.24	0.12	0.060	0.030	0.015	0.0075

##### 2.3.1.3 Preparation of LPS solution

We dissolved 10 mg of LPS in 10 ml of PBS to obtain LPS stock solution at a concentration of 1 mg/ml. The solution was filtered through a 0.22-μm microporous membrane and stored at −20°C. The solution was diluted with complete medium to the desired concentration before use.

##### 2.3.1.4 Preparation of MTS solution

The appropriate amount of MTS was dissolved in DMEM culture solution at a ratio of 1:20, avoiding light.

##### 2.3.1.5 *I. cappa* extract stock solution for cells

First, 5 g of *I. cappa* extract was dissolved in DMSO by ultrasonication. PBS was added to a final volume of 25 ml to obtain a concentration of 200 mg/ml. The extract was filtered through a 0.22-μm microporous membrane and stored in aliquots at −20°C. Stock solution was diluted to the desired concentration with complete medium before use.

#### 2.3.2 Establishment of inflammatory cell model

##### 2.3.2.1 Safe concentration range of LPS in RAW 264.7 cells

RAW264.7 cells were cultured in DMEM with 10% FBS at 37 °C and 5% CO_2_ for 36–48 h with 1:6 passaging. When the cells grew to 90% confluency, we digested them with 0.25% trypsin, adjusted the cell density to 1 × 10^5^ cells/mL, inoculated them in 96-well culture plates (100 μL per well), and incubated them at 37°C for 24 h. The following groups were set up: the normal control group (100 μL of DMEM with 10% FBS in each well) and the model group (100 μL of DMEM with 10% FBS containing 0.1, 0.2, 0.5, 1, or 2 μg/ml LPS in each well). After 24 h of incubation, cell activity was measured by the MTS method. The experiment was performed in six parallel wells per concentration and repeated three times. Cell viability was calculated as follows:

$$\text{ Cell viability}\left(\%\right)=\frac{\text{OD}\left(\text{experimental group}\right)}{\text{OD}\left(\text{blank group}\right)}\times 100\%$$

##### 2.3.2.2 Screening of LPS concentration and incubation time in RAW 264.7 cells

Cells at the logarithmic growth stage were selected for counting. The cell suspension was adjusted to 1 × 10^5^ cells/well, and cells were inoculated in 6-well plates (2 ml of cell suspension per well). The following groups were set up: the normal control group (DMEM with 10% FBS in each well) and the model group (DMEM with 10% FBS containing 0.1, 0.2, 0.5, 1, or 2 μg/ml LPS). The supernatant was collected after 6, 12, and 24 h of incubation, and nitric oxide (NO) and tumor necrosis factor-α (TNF-α) levels were measured using commercial kits.

##### 2.3.2.3 Safe concentration range of *I. cappa* extract in RAW 264.7 cells

Cells at the logarithmic growth stage were trypsinized, and the cell suspension was adjusted to 1 × 10^5^ cells/well. Cells were inoculated in a 96-well plate (100 μL per well) and incubated for 24 h. The medium was discarded, the cells were washed twice with PBS buffer, and 100 μL of DMEM containing 10% FBS and different concentrations of *I. cappa* extract (0, 100, 200, 400, 600, 800, 1000, 2000, and 4000 μg/ml) was added.

Six replicate wells were set up and incubated for 24 h. The medium was discarded, and the cells were washed three times with PBS buffer and then incubated for 2–4 h with ready-made MTS solution. We measured the OD value at 490 nm and calculated the cell survival rate according to the above equation.

#### 2.3.3 Cellular pharmacokinetic experiments of five active ingredients after treatment with 200 μg/ml and 800 μg/ml of *I. cappa* extracts

Cells at the logarithmic growth stage were trypsinized, and the cell suspension was adjusted to 1 × 10^5^ cells/well. Cells were inoculated in 6-well plates (2 ml of cell suspension per plate). The following groups were set up: the normal group (no LPS and zero, 200 μg/ml, and 800 μg/ml *I. cappa* extract concentrations) and the model group (LPS and zero, 200 μg/ml, and 800 μg/ml *I. cappa* extract concentrations). After 24 h, the medium was discarded. We added 2 ml of DMEM containing 10% FBS per well to the normal group and 2 ml of DMEM containing 10% FBS and 1 μg/ml LPS per well to the model group. Both groups were incubated for 24 h, the medium was discarded, and the cells were washed twice with PBS. The dosing settings were as follows: DMEM containing 10% FBS in the normal groups; DMEM containing 10% FBS and 200 μg/ml of *I. cappa* extract (an equivalence of 0.80 mmol/L LUT, 1.47 mmol/L CA, 1.69 mmol/L CCA, 17.5 mmol/L 3,4-DCQA, and 16.88 mmol/L 4,5-DCQA); and DMEM containing 10% FBS and 800 μg/ml of *I. cappa* extract (an equivalence of 3.20 mmol/L LUT, 5.88 mmol/L CA, 6.76 mmol/L CCA, 70.00 mmol/L 3,4-DCQA, and 67.52 mmol/L 4,5-DCQA). Cells were collected at 0, 5, 15, 30, 45 min, 1, 2, 4, 6, 8, 12, and 24 h. Cells were washed twice quickly with 2 ml of pre-chilled PBS, and 2 ml of ultrapure water was added to each well. The cells were frozen and thawed at −80°C three times before collecting the dissociated cells from the 6-well plates into centrifuge tubes for cellular pharmacokinetic studies.

A previously established UPLC-MS/MS analytical method was applied to determine the intracellular concentrations of the five ingredients in *I. cappa* extract (Zhou, et al., 2022)*.* Briefly, puerarin was selected as an internal standard and the positive and negative ion modes were used for simultaneous monitoring. Quantitation was performed using multiple-reaction monitoring of the protonated molecular ion to the predominant product ion pair (m/z 449.2 > 287.1 for LUT and m/z 417.0 > 267.0 for puerarin detected in the positive ion mode; m/z 353.1 > 191.1 for CA, m/z 353.2 > 172.9 for CCA, m/z 515.1 > 353.1 for 3,4-DCQA, and m/z 515.1 > 353.1 for 4,5-DCQA in the negative ion mode). An aliquot of 800 μL cell suspension after three freeze–thaw cycles was spiked with 50 μL of the internal standard solution containing 0.036 μmol/L puerarin and vortexed briefly. Then, the mixture was added to 160 μL of aqueous 20% formic acid and 800 μL of methanol, followed by 5 min of vortexing and 10 min of sonication. The supernatant was centrifuged at 12,000 rpm for 10 min at 4°C and blown dry using a nitrogen blower. Next, 150 μL of 50% methanol was added to the residue, and samples were vortexed for 5 min, sonicated for 10 min, and centrifuged at 14,000 rpm for 10 min at 4°C. The supernatant was transferred into an autosampler vial, and an aliquot of 3 μL was subsequently injected into the UPLC-MS/MS system for assay. The lower limit of quantification was established at 0.0011 μmol/L for LUT, 0.0027 μmol/L for CA and CCA, and 0.0074 μmol/L for 3,4-DCQA and 4,5-DCQA in cell suspension.

#### 2.3.4 BCA protein quantification

We used BCA kits to determine protein concentrations.

#### 2.3.5 Data analysis

SPSS18.0 statistical software was used for data analysis, and experimental results were expressed as mean ± standard deviation. We used the independent samples *t*-test for comparisons between groups, and *p* < 0.05 was considered statistically different. Pharmacokinetic parameters were processed with WinNonLin 8.3 data processing software.

## 3 Results

### 3.1 *In vitro* inflammation model validation

#### 3.1.1 Cell morphology

**FIGURE 2:**
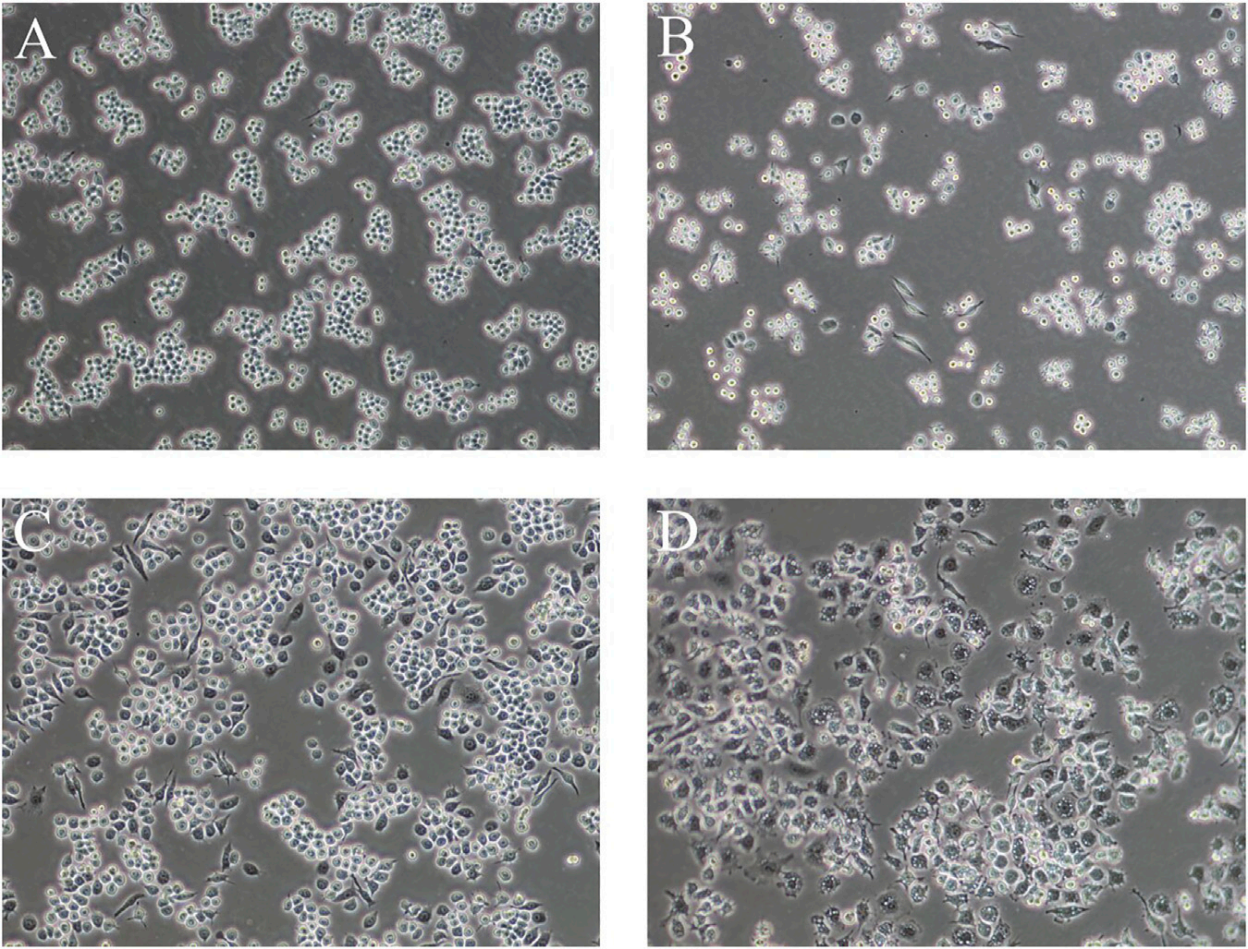
Cell morphology of LPS-induced RAW264.7 cells at different times **(A)**: normal cells, **(B)** 1 μg/ml LPS-induced cells at 6 h, **(C)** 1 μg/ml LPS-induced cells at 12 h, **(D)** 1 μg/ml LPS-induced cells at 24 h) (100×).

As shown in Figure 2, the control RAW264.7 cells were small, round, and translucent, showed low differentiation, and had no pseudopods. Upon LPS induction, the morphology of the cells changed significantly: the cells were larger, the intracellular vacuoles were obvious, the pseudopods were longer and more extended, and the cells were irregularly spindle-shaped and polygonal. This change in morphology became larger with longer induction times.

#### 3.1.2 Survival rate of cells treated with different concentrations of LPS after 24 h

**FIGURE 3:**
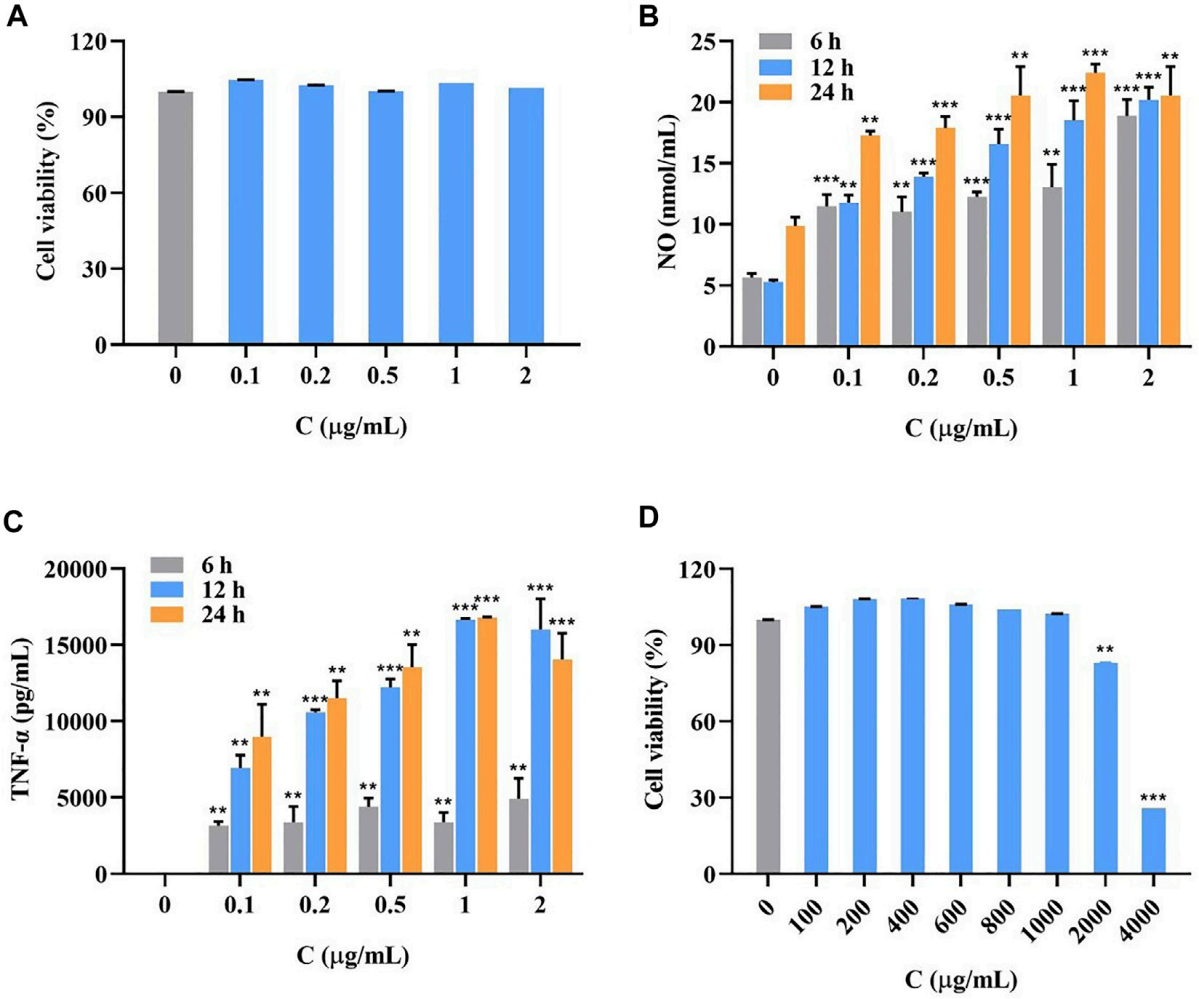
Survival rate of LPS-induced RAW264.7 cells at LPS different concentrations for 24 h **(A)**, the accumulation of NO **(B)** and TNF-α **(C)** in LPS-induced RAW264.7 cells at different concentrations for 6, 12 and 24 h ( $\bar{x}$ ± SD, *n* = 3, nmol/mL), and survival rate of RAW264.7 cells after the addition of *I. cappa* extract **(D)**. Compared with the blank group, *p < 0.05, **p < 0.01, ***p < 0.001.

Cells were treated with different concentrations of LPS. As shown in Figure 3A, after 24 h, the cell survival rate was not significantly affected.

#### 3.1.3 Measurement of inflammatory factor secretion

As shown in Figure 3, the secretion of inflammatory factors NO (Figure 3B) and TNF-α (Figure 3C) significantly increased with longer LPS treatment and higher concentrations. The secretion was most pronounced at an induction time of 24 h and a concentration of 1 μg/ml. The data of all groups were significantly different compared with the blank group.

#### 3.1.4 Safe concentration range of *I. cappa* in RAW264.7 cells

As shown in Figure 3D, the cell survival rate was above 90% at *I. cappa* extract concentrations of 0–1000 μg/ml, indicating that the drug was not significantly toxic to the cells. Considering this safe range and the detectability of trace amounts of drug entering cells, the concentrations of 200 and 800 μg/ml were chosen for the pharmacokinetic study.

### 3.2 Pharmacokinetic results

After calculating the measured intracellular concentrations of the five ingredients in *I. cappa* extract, the pharmacokinetic software WinNonlin 8.3 (Phoenix, Pharsight, United States) was used to plot the mean cell concentration (ng/mg) versus time profile for the above drug components. Non-compartmental analysis was performed to find the pharmacokinetic parameters after treatment with 200 μg/ml and 800 μg/ml of *I. cappa* extracts, including terminal elimination half-life (T_1/2_), area under the concentration versus time curve from zero to last sampling time (AUC_0–t_), volume of distribution (V_d_/F), and total body clearance (CL/F). The peak concentration (C_max_) and the time to reach C_max_ (T_max_) were read directly from the individual concentration-time data. The pharmacokinetic parameters of the five representative ingredients of *I. cappa* were compared and analyzed between the normal and the model groups at low and high concentrations to illustrate the kinetic process of the drug in the cells.

**FIGURE 4:**
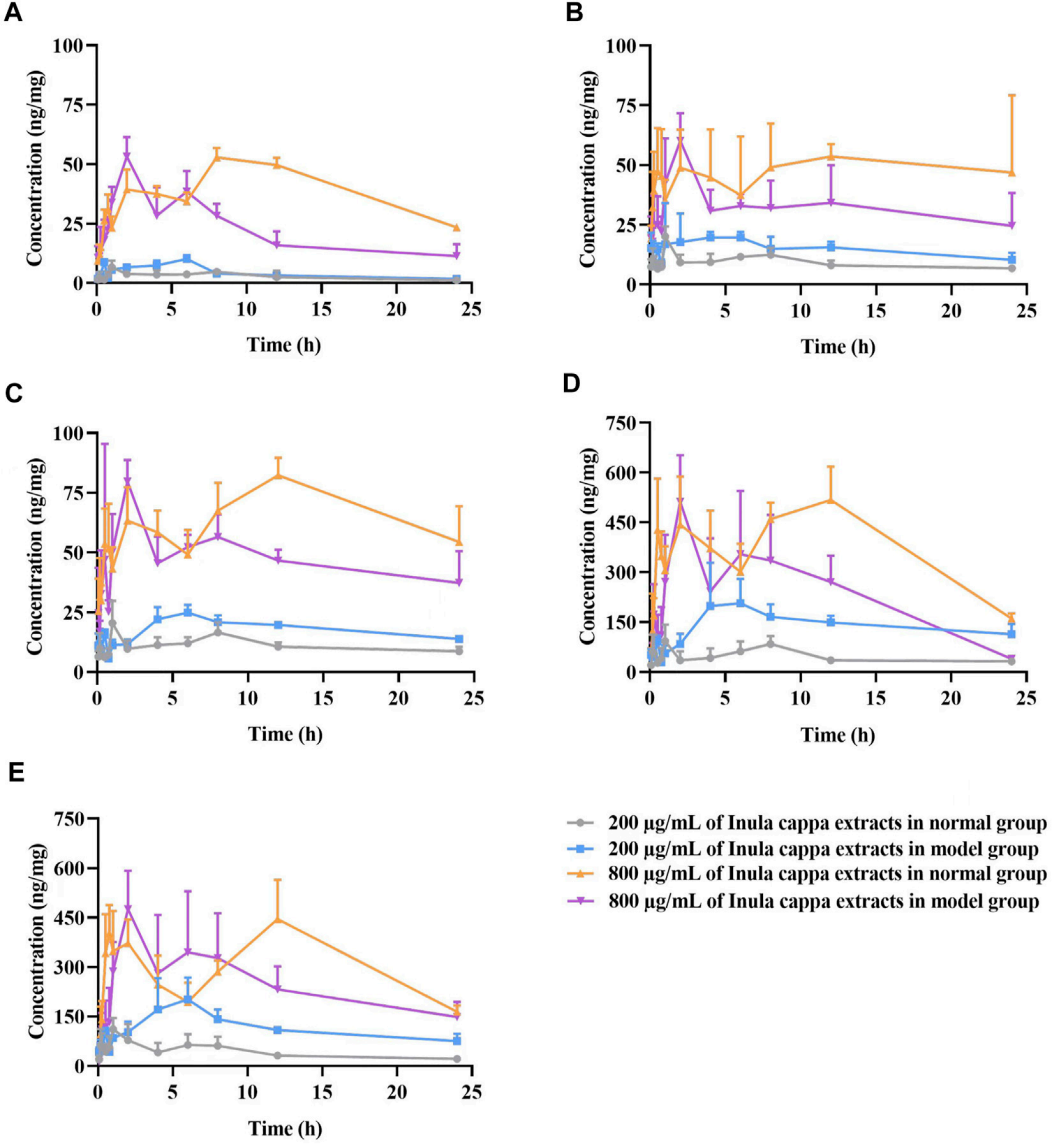
The mean concentration (ng/mg) of luteolin **(A)**, chlorogenic acid **(B)**, cryptochlorogenic acid **(C)**, 3,4-dicaffeoylquinic acid **(D)**, and 4,5-dicaffeoylquinic acid **(E)** versus time profiles in RAW264.7 cells after treatment with 200 μg/ml and 800 μg/ml of *I. cappa* extracts ( $\bar{x}$ ± SD, *n* = 6).

For LUT, as shown in Figure 4A and Table 2, the AUC_0-t_ and C_max_ values of the main pharmacokinetic parameters of LUT in normal and inflammatory RAW264.7 cells after administration of *I. cappa* extract showed a dose-dependent effect. With increasing doses, C_max_ and AUC also tended to increase, reflecting a dose-dependent pharmacokinetic profile. However, there were differences in their T_max_, CL_F, Vz_F, and MRT_0-t_ values among them. After treatment with 200 μg/ml of *I. cappa* extracts, in normal cells, the AUC_(0-t)_, T_max_, and CL_F values were 66.85 ± 25.23 h*ng/mL, 1.20 ± 0.45 h, and 8.64 ± 2.64 ml/h, respectively, while in inflammatory cells, the parameters were 99.66 ± 21.24 h*ng/mL, 6.00 ± 0.00 h, and 5.73 ± 0.96 ml/h, respectively. The results showed that the uptake of LUT in inflammatory cells was faster at low concentrations, but T_max_ lagged and uptake of LUT was slower than in the normal group, and CL_F and Vz_F were also lower than in the normal group. After treatment with 800 μg/ml of *I. cappa* extracts, in normal cells, the AUC_0–t_, T_max_, Vz_F, and CL_F values were 931.01 ± 14.06 h*ng/mL, 10.00 ± 2.19 h, 36.76 ± 2.76 ml, and 2.09 ± 0.06 ml/h, respectively, while in inflammatory cells, the corresponding parameters were 528.65 ± 82.74 h*ng/mL, 2.00 ± 0.00 h, 61.50 ± 12.67 ml, and 4.10 ± 1.49 ml/h, respectively. The results show that after treatment with 800 μg/ml of *I. cappa* extracts, the uptake of LUT in inflammatory cells was slower, but T_max_ was prolonged, absorption was faster, t_1/2_ was shorter, metabolism was prolonged, and CL_F was increased. The pharmacokinetic parameters of LUT at each concentration differed significantly between the normal and inflammatory states.

**TABLE 2:** The pharmacokinetic parameters of luteolin in RAW264.7 cells after treatment with 200 μg/ml and 800 μg/ml of *I. cappa* extracts ( $\bar{x}$ ± SD, n = 6).

Parameters	unit	200 μg/ml		800 μg/ml	
		Normal group	Model group	Normal group	Model group
AUC_0-t_	h*ng/mL	66.85 ± 25.23	99.66 ± 21.24*	931.01 ± 14.06	528.65 ± 82.74***
AUC_0-∞_	h*ng/mL	86.61 ± 23.13	125.30 ± 22.12*	1343.24 ± 36.14	736.26 ± 175.54***
MRT_0-t_	h	9.05 ± 0.48	8.57 ± 0.41	11.01 ± 0.28	8.82 ± 0.91**
MRT_0-∞_	h	16.73 ± 4.70	14.86 ± 2.67	20.44 ± 1.18	17.54 ± 5.13
t_1/2_	h	10.85 ± 3.07	10.09 ± 1.83	12.24 ± 1.21	11.50 ± 3.95
T_max_	h	1.20 ± 0.45	6.00 ± 0.00***	10.00 ± 2.19	2.00 ± 0.00***
CL_F	mL/h	8.64 ± 2.64	5.73 ± 0.96*	2.09 ± 0.06	4.10 ± 1.49*
Vz_F	mL	134.94 ± 48.39	83.95 ± 21.53	36.76 ± 2.76	61.50 ± 12.67**
C_max_	ng/mL	8.11 ± 1.42	10.14 ± 1.55*	54.19 ± 2.37	53.16 ± 8.22
*R* ^2^	-	0.70 ± 0.09	0.90 ± 0.04	0.96 ± 0.02	0.90 ± 0.13

Compared with normal group, *p < 0.05, **p < 0.01, ***p < 0.001.

For CA, as shown in Figure 4B and Table 3, in normal and inflammatory RAW264.7 cells, the AUC_0-t_ and C_max_ values of CA after administration of *I. cappa* extract showed a dose-dependent effect. The C_max_ and AUC values tended to increase with increasing concentrations. After treatment with 200 μg/ml of *I. cappa* extracts, in control cells, the AUC_0-t_, T_max_, C_max_, CL_F, and Vz_F values were 219.14 ± 19.64 h*ng/mL, 1.00 ± 0.00 h, 20.08 ± 4.17 ng/ml, 2.56 ± 0.46 ml/h, and 72.09 ± 8.62 ml, respectively, while in inflammatory cells, the corresponding parameters were 405.87 ± 153.61 h*ng/mL, 3.40 ± 1.95 h, 33.62 ± 14.14 ng/ml, 1.62 ± 0.48 ml/h, and 41.55 ± 11.87 ml, respectively. The results showed that CA uptake in inflammatory cells was faster after treatment with 200 μg/ml of *I. cappa* extracts, but T_max_ lagged and CA uptake was slower than in the normal group, and CL_F and Vz_F were also lower than in the normal group. The AUC_0–t_, T_max_, t_1/2_, and Vz_F values of CA in normal cells After treatment with 800 μg/ml of *I. cappa* extracts were 1151.63 ± 260.90 h*ng/mL, 13.00 ± 9.44 h, 12.62 ± 1.99 h, and 45.10 ± 14.67 ml, respectively, and the corresponding parameters in inflammatory cells were 715.66 ± 160.49 h*ng/mL, 2.00 ± 0.00 h, 20.57 ± 6.79 h, and 93.61 ± 16.19 ml, respectively. The results showed that after treatment with 800 μg/ml of *I. cappa* extracts, CA uptake in inflammatory cells was slower than that in normal cells, but T_max_ was prolonged, uptake was faster, t_1/2_ was prolonged, metabolism was slower, and CL_F and Vz_F increased. The pharmacokinetic parameters of CA at each concentration differed significantly between normal and inflammatory states.

**TABLE 3:** The pharmacokinetic parameters of chlorogenic acid in RAW264.7 cells after treatment with 200 μg/ml and 800 μg/ml of *I. cappa* extracts ( $\bar{x}$ ± SD, n = 6).

Parameters	unit	200 μg/ml		800 μg/ml	
		Normal group	Model group	Normal group	Model group
AUC_0-t_	h*ng/mL	219.14 ± 19.64	405.87 ± 153.61*	1151.63 ± 260.90	715.66 ± 160.49**
AUC_0-∞_	h*ng/mL	419.19 ± 77.98	695.12 ± 215.82*	1881.18 ± 846.92	1377.31 ± 610.95
MRT_0-t_	h	10.63 ± 0.60	10.74 ± 0.97	11.95 ± 1.87	10.22 ± 0.96
MRT_0-∞_	h	30.81 ± 7.04	27.99 ± 10.99	22.75 ± 4.28	30.26 ± 9.96
t_1/2_	h	20.12 ± 4.79	18.56 ± 6.82	12.62 ± 1.99	20.57 ± 6.79*
T_max_	h	1.00 ± 0.00	3.40 ± 1.95**	13.00 ± 9.44	2.00 ± 0.00*
CL_F	mL/h	2.56 ± 0.46	1.62 ± 0.48**	2.48 ± 0.75	3.47 ± 1.30
Vz_F	mL	72.09 ± 8.62	41.55 ± 11.87**	45.10 ± 14.67	93.61 ± 16.19***
C_max_	ng/mL	20.08 ± 4.17	33.62 ± 14.14*	53.46 ± 18.86	60.10 ± 11.63
*R* ^2^	-	0.67 ± 0.23	0.77 ± 0.14	0.80 ± 0.23	0.78 ± 0.15

Compared with normal group, *p < 0.05, **p < 0.01, ***p < 0.001.

For CCA, as shown in Figure 4C and Table 4, the AUC _0–t_ and C_max_ values of CCA in normal and inflammatory RAW264.7 cells after administration of *I. cappa* extract showed a dose-dependent effect. The C_max_ and AUC values tended to increase with increasing concentrations. After treatment with 200 μg/ml of *I. cappa* extracts, the AUC_0-t_, T_max_, CL_F, and Vz_F values in normal cells were 265.24 ± 31.31 h*ng/mL, 1.00 ± 0.00 h, 2.49 ± 0.50 ml/h, and 70.37 ± 16.71 ml, respectively, while in inflammatory cells, the corresponding parameters were 427.29 ± 32.92 h*ng/mL, 6.33 ± 0.82 h, 1.32 ± 0.23 ml/h, and 45.98 ± 6.16 ml, respectively. The results showed that after treatment with 200 μg/ml of *I. cappa* extracts, CCA uptake in inflammatory cells increased, but T_max_ lagged and the uptake of CCA in the inflammatory cell group was slower than that in the normal group, and CL_F and Vz_F were also lower than in the normal group. After treatment with 800 μg/ml of *I. cappa* extracts, the AUC_0-t_ and T_max_ values in normal cells were 1559.95 ± 139.88 h*ng/mL and 11.33 ± 1.63 h, respectively, while the corresponding parameters in inflammatory cells were 1138.24 ± 70.46 h*ng/mL and 1.75 ± 0.61 h, respectively. The results showed that after treatment with 800 μg/ml of *I. cappa* extracts, CCA uptake in inflammatory cells was reduced, T_max_ was prolonged, the uptake of CCA was faster, t_1/2_ was prolonged, metabolism was slowed down, and Vz_F and CL_F were increased. The pharmacokinetic parameters of CCA differed significantly between normal and inflammatory states at each concentration.

**TABLE 4:** The pharmacokinetic parameters of cryptochlorogenic acid in RAW264.7 cells after treatment with 200 μg/ml and 800 μg/ml of *I. cappa* extracts ( $\bar{x}$ ± SD, n = 6).

Parameters	unit	200 μg/ml		800 μg/ml	
		Normal group	Model group	Normal group	Model group
AUC_0-t_	h*ng/mL	265.24 ± 31.31	427.29 ± 32.92***	1559.95 ± 139.88	1138.24 ± 70.46***
AUC_0-∞_	h*ng/mL	495.91 ± 92.60	928.16 ± 168.41***	2823.69 ± 669.23	2420.18 ± 1003.94
MRT_0-t_	h	11.04 ± 0.74	11.42 ± 0.42	11.91 ± 0.72	10.91 ± 0.87
MRT_0-∞_	h	30.25 ± 8.70	37.64 ± 9.46	38.74 ± 23.85	37.01 ± 23.93
t_1/2_	h	19.39 ± 6.15	24.89 ± 6.53	18.10 ± 5.71	23.76 ± 18.54
T_max_	h	1.00 ± 0.00	6.33 ± 0.82***	11.33 ± 1.63	1.75 ± 0.61***
CL_F	mL/h	2.49 ± 0.50	1.32 ± 0.23**	1.56 ± 0.59	2.29 ± 0.98
Vz_F	mL	70.37 ± 16.71	45.98 ± 6.16**	43.99 ± 3.28	59.93 ± 26.43
C_max_	ng/mL	21.90 ± 7.29	25.32 ± 3.26	84.79 ± 5.92	89.04 ± 26.15
*R* ^2^	-	0.84 ± 0.31	0.69 ± 0.05	0.74 ± 0.19	0.94 ± 0.05

Compared with normal group, *p < 0.05, **p < 0.01, ***p < 0.001.

For 3,4-DCQA, as shown in Figure 4D and Table 5, the AUC_0–t_ and C_max_ values of 3,4-DCQA in cells after the administration of *I. cappa* extract to normal and inflammatory RAW 264.7 cells showed a dose-dependent effect. The C_max_ and AUC values tended to increase with increasing concentrations. After treatment with 200 μg/ml of *I. cappa* extracts, in normal cells, the AUC_0-t_, T_max_, C_max_, MRT_0–t_, t_1/2_, CL_F, and Vz_F values were 1168.85 ± 240.66 h*ng/mL, 1.00 ± 0.00 h, 136.24 ± 70.99 ng/ml, 10.40 ± 0.74 h, 13.32 ± 2.70 h, 10.59 ± 2.30 ml/h, and 201.47 ± 52.06 ml, respectively, while in inflammatory cells, the corresponding parameters were 3499.51 ± 536.34 h*ng/mL, 6.00 ± 3.10 h, 255.49 ± 79.43 ng/ml, 11.49 ± 0.90 h, 20.32 ± 4.09 h, 2.77 ± 0.68 ml/h, and 77.99 ± 10.00 ml, respectively. The results showed that after treatment with 200 μg/ml of *I. cappa* extracts, 3, 4-DCQA uptake was increased in inflammatory cells, but T_max_ lagged, its uptake was slower compared to the normal group, t_1/2_ was prolonged, metabolism was slowed down, and CL_F and Vz_F were reduced. After treatment with 800 μg/ml of *I. cappa* extracts, in normal cells, the AUC_0–t_, T_max_, t_1/2_, Vz_F, and MRT_0–∞_ values of 3,4-DCQA were 8916.50 ± 1010.81 h*ng/mL, 9.00 ± 4.69 h, 8.41 ± 2.10 h, 80.82 ± 18.46 ml, and 15.11 ± 1.69 h, respectively, and the corresponding parameters in inflammatory cells were 6426.51 ± 1438.14 h*ng/mL, 2.50 ± 1.76 h, 16.30 ± 6.63 h, 147.13 ± 20.73 ml, and 24.50 ± 9.39 h, respectively. The results showed that after treatment with 800 μg/ml of *I. cappa* extracts, 3,4-DCQA uptake in inflammatory cells was reduced, T_max_ was advanced, absorption was faster, t_1/2_ was prolonged, metabolism was slowed, and Vz_F was increased. Significant differences were observed in the pharmacokinetic parameters of 3, 4-DCQA at various concentrations between normal and inflammatory states.

**TABLE 5:** The pharmacokinetic parameters of 3,4-dicaffeoylquinic acid in RAW264.7 cells after treatment with 200 μg/ml and 800 μg/ml of *I. cappa* extracts ( $\bar{x}$ ± SD, n = 6).

Parameters	unit	200 μg/ml		800 μg/ml	
		Normal group	Model group	Normal group	Model group
AUC_0-t_	h*ng/mL	1168.85 ± 240.66	3499.51 ± 536.34***	8916.50 ± 1010.81	6426.51 ± 1438.14**
AUC_0-∞_	h*ng/mL	1776.35 ± 384.24	6926.13 ± 1912.52***	10884.10 ± 1066.05	11408.00 ± 3813.22
MRT_0-t_	h	10.40 ± 0.74	11.49 ± 0.90*	10.33 ± 0.44	10.54 ± 1.02
MRT_0-∞_	h	21.69 ± 4.31	31.85 ± 6.76*	15.11 ± 1.69	24.50 ± 9.39*
t_1/2_	h	13.32 ± 2.70	20.32 ± 4.09**	8.41 ± 2.10	16.30 ± 6.63*
T_max_	h	1.00 ± 0.00	6.00 ± 3.10**	9.00 ± 4.69	2.50 ± 1.76**
CL_F	mL/h	10.59 ± 2.30	2.77 ± 0.68***	6.70 ± 0.66	6.87 ± 2.04
Vz_F	mL	201.47 ± 52.06	77.99 ± 10.00***	80.82 ± 18.46	147.13 ± 20.73***
C_max_	ng/mL	136.24 ± 70.99	255.49 ± 79.43*	579.51 ± 100.02	612.61 ± 147.92
*R* ^2^	-	0.91 ± 0.38	0.67 ± 0.36	0.93 ± 0.09	0.86 ± 0.12

Compared with normal group, *p < 0.05, **p < 0.01, ***p < 0.001.

For 4,5-DCQA, as shown in Figure 4E and Table 6, the AUC_0–t_ and C_max_ values of 4,5-DCQA in normal and inflammatory RAW264.7 cells after administration of *I. cappa* extract showed a dose-dependent effect. The C_max_ and AUC values tended to increase with increasing concentrations. After treatment with 200 μg/ml of *I. cappa* extracts, the AUC_0–t_, T_max_, MRT_0–t_, t_1/2_, CL_F, and Vz_F values in normal cells were 1032.84 ± 280.12 h*ng/mL, 0.96 ± 0.60 h, 10.44 ± 2.39 h, 8.86 ± 0.76 h, 13.98 ± 4.51 ml/h, and 201.17 ± 42.85 ml, respectively, while in inflammatory cells, the corresponding parameters were 2817.10 ± 572.08 h*ng/mL, 5.67 ± 1.51 h, 10.43 ± 0.41 h, 15.05 ± 1.10 h, 4.08 ± 0.87 ml/h, and 88.80 ± 20.50 ml, respectively. The results showed that after treatment with 200 μg/ml of *I. cappa* extracts, 4,5-DCQA uptake was increased in inflammatory cells, but its T_max_ was lagged, uptake was slower than in the normal group, t_1/2_ and MRT were prolonged, metabolism was slowed, and CL_F and Vz_F were also reduced. After treatment with 800 μg/ml of *I. cappa* extracts, the AUC_0–t_, T_max_, t_1/2_, and Vz_F values of 4,5-DCQA in normal cells were 7475.39 ± 996.99 h*ng/mL, 7.00 ± 5.48 h, 9.09 ± 2.69 h, and 94.80 ± 24.43 ml, respectively, and the corresponding parameters in inflammatory cells were 5955.63 ± 1217.95 h*ng/mL, 2.50 ± 1.76 h, 12.25 ± 3.62 h, and 136.49 ± 18.20 ml, respectively. The results showed that 4,5-DCQA uptake in inflammatory cells was reduced, T_max_ was prolonged, its uptake was faster, t_1/2_ was prolonged, metabolism was slowed down, and Vz_F and CL_F were increased after treatment with 800 μg/ml of *I. cappa* extracts. Significant differences were observed in the pharmacokinetic parameters of 4,5-DCQA at various concentrations between normal and inflammatory states.

**TABLE 6:** The pharmacokinetic parameters of 4,5-dicaffeoylquinic acid in RAW264.7 cells after treatment with 200 μg/ml and 800 μg/ml of *I. cappa* extracts ( $\bar{x}$ ± SD, n = 6).

Parameters	unit	200 μg/ml		800 μg/ml	
		Normal group	Model group	Normal group	Model group
AUC_0-t_	h*ng/mL	1032.84 ± 280.12	2817.10 ± 572.08***	7475.39 ± 996.99	5955.63 ± 1217.95*	
AUC_0-∞_	h*ng/mL	1361.08 ± 417.72	4448.02 ± 989.58***	9672.25 ± 983.10	8967.64 ± 2062.61	
MRT_0-t_	h	8.86 ± 0.76	10.43 ± 0.41*	10.62 ± 0.68	10.19 ± 1.41	
MRT_0-∞_	h	16.03 ± 2.59	23.27 ± 1.68***	16.84 ± 3.27	18.74 ± 4.75	
t_1/2_	h	10.44 ± 2.39	15.05 ± 1.10**	9.09 ± 2.69	12.25 ± 3.62*	
T_max_	h	0.96 ± 0.60	5.67 ± 1.51**	7.00 ± 5.48	2.50 ± 1.76*	
CL_F	mL/h	13.98 ± 4.51	4.08 ± 0.87***	7.28 ± 0.71	8.11 ± 1.80	
Vz_F	mL	201.17 ± 42.85	88.80 ± 20.50***	94.80 ± 24.43	136.49 ± 18.20**	
C_max_	ng/mL	153.88 ± 59.85	224.23 ± 60.39	575.66 ± 159.23	589.11 ± 148.35	
*R* ^2^	-	0.90 ± 0.12	0.66 ± 0.15	1.00 ± 0.00	0.92 ± 0.08	

Compared with normal group, *p < 0.05, **p < 0.01, ***p < 0.001.

## 4 Discussion

The LPS-induced *in vitro* inflammation model is a commonly used research tool for natural drug screening. This cell model is relatively simple, rapid, sensitive, and efficient and requires only small amounts of sample. We observed that after modeling, cells were larger, the intracellular vacuole was obvious, the pseudopods were longer and more extended, and the cells were more irregularly spindle-shaped and polygonal. According to the literature, RAW264.7 cells in this form are in a state of inflammation (Zhou, et al., 2019; Zhang, et al., 2020), and this morphological change was more significant with increasing induction time. The secretion of NO and TNF-α increased with the increase of LPS induction time and concentration. Based on our results and the literature, we chose an induction time of 24 h and a concentration of 1 μg/ml for the establishment of the *in vitro* inflammation model.

The cellular pharmacokinetic results showed that the uptake of five ingredients (LUT, CA, CCA, 3,4-DCQA, and 4,5-DCQA) by RAW264.7 cells is a nonlinear pharmacokinetic process. Their pharmacokinetic parameters changed with concentration; C_max_ and AUC kinetics were not proportional to the concentration, and t_1/2_ was prolonged with increasing concentrations. For LUT and CCA, their C_max_ and AUC values increased with increasing concentrations and were not proportional in both normal and model cells, which indicates that the cellular uptake of the two ingredients was concentration-dependent. The differences in C_max_ and AUC of CA were not significant after treatment with 200 μg/ml of *I. cappa* extracts, even in inflammatory cells, where both C_max_ and AUC increased substantially after treatment with 800 μg/ml of *I. cappa* extracts. In normal cells 3,4-DCQA and 4,5-DCQA showed a concentration-dependent increase. Overall, the AUC values of the five components at different concentrations were not proportional to the administered concentrations and exhibited a nonlinear pharmacokinetic process.

In normal cells, t_1/2_ of LUT was prolonged with increasing concentrations, but there was no significant difference. The t_1/2_ values of CA and CCA were shorter with increasing concentrations, and the t_1/2_ values of 3,4-DCQA and 4,5-DCQA remained essentially unchanged with increasing concentrations. In inflammatory cells, the t_1/2_ values of the remaining four components were prolonged with increasing concentrations, except for LUT, whose t_1/2_ became shorter and metabolism accelerated with an increasing concentration. Moreover, T_max_ of the five ingredients in inflammatory cells was prolonged after treatment with 200 μg/ml of *I. cappa* extracts, showing that the uptake was slower than in the normal group, and the T_max_ values of each component in normal and inflammatory cells were inconsistent. However, t_1/2_ was prolonged, metabolism slowed down, and both CL_F and Vz_F decreased significantly. After treatment with 800 μg/ml of *I. cappa* extracts, compared to the normal cell group, T_max_ of the components was prolonged and absorption became faster in the model group. This indicates that the morphology and internal microenvironment of the cells are altered, except in the inflammatory state. This leads to differences in the entry of the drug into the body, and we speculate that this difference may result from the increase in concentration.

We previously determined that the plasma protein binding abilities of LUT, CA, CCA, 3,4-DCQA, and 4,5-DCQA in human and rat plasma were in the range of 81.25 ± 0.038% to 97.46 ± 0.013% by equilibrium dialysis (Bao, et al., 2019). This indicates that all five components have strong protein binding abilities. For this type of drug, at increasing drug doses, plasma protein binding is decreased, the free drug concentration is increased, and absorption distribution and pharmacological action are altered (Feng, et al., 2019). In addition, FBS contains a large number of proteins, and it has been shown that polyphenolic compounds, like bovine serum albumin and globulin, can interact with each other (Mai, et al., 2021). According to the nonlinear pharmacokinetic characteristics, the metabolic pathways and elimination processes were facilitated and the half-life was decreased when we increased the dose of components with strong plasma protein binding ability. This could explain why there was a difference in t_1/2_, CL_F, and other values for each component between different concentrations.

Drug absorption is related to drug physicochemical properties, cell membrane permeability, and drug transporters. Biological membrane permeation is a key factor for the cellular uptake of free drugs. The cell membrane is selectively permeable and has a bilayer lipid structure. Thus, selectively absorbed ions and small molecules and lipid-soluble drugs can easily pass through the cell membrane. Generally, some drugs with low lipid solubility, large molecular weight, and polar groups enter cells by endocytosis (Xie, et al., 2004). CA is a caffeic acid with high polarity, and macrophages may take up the drug into the cells by endocytosis. The change of cell permeability is an important pathological process in a variety of diseases such as inflammatory responses, tissue hypoxia, and impaired cellular metabolism (Zhang, et al., 2018). Cellular modeling causes an increase in permeability, which is presumed to be associated with increased p38 MAPK pathway and PI3K/Akt pathway activity during LPS-induced production of inflammatory factors, according to the relevant literature (Chang and Qi, 2020). Macrophages are immune cells that rely on their phagocytosis and clearance ability to participate in the immune process. They have a variety of receptors on their surface that are involved in cytophagy or cytokinesis. During LPS treatment, LPS combines with Toll-like receptor 4 proteins on the cell surface, actin aggregates, the cytoskeleton contracts, and pseudopods are extended. At this time, the phagocytic capacity of RAW264.7 cells is greatly enhanced (Wang, et al., 2001), which causes the cells to swallow particles from the external environment into the cells, increasing drug uptake.

In general, the differences in the pharmacokinetic parameters of different concentration compounds between the normal and model groups were inconsistent. After treatment with 200 μg/ml of *I. cappa* extracts, the AUC_0-t_, MRT_0-t_, T_max_, and C_max_ values of the five compounds in the model group were higher than those in the normal group, while the CL_F and Vz_F values in the model group were lower than those in the normal group; After treatment with 800 μg/ml of *I. cappa* extracts, their AUC_0-t_, MRT_0-t_, T_max_, and C_max_ values in the model group were lower than those in the normal group, while CL_F and Vz_F values in the model group were higher than those in the normal group. These results indicated that compared with the normal group, the five compounds in the model group had increased absorption and slowed elimination after treatment with 200 μg/ml of *I. cappa* extracts, while the absorption decreased and elimination accelerated in the model group after treatment with 800 μg/ml of *I. cappa* extracts. The main reason for this phenomenon is that the permeability of the cell membrane increases in the inflammatory state. Therefore, after treatment with 200 μg/ml of *I. cappa* extracts, the absorption of the drug in the model group was increased. However, after treatment with 800 μg/ml of *I. cappa* extracts, the AUC values of five ingredients were reduced in the model group compared with the normal group. We hypothesize that this might be explained as follows. First, drug toxicity may play a role. The composition of *I. cappa* extract is complex. With the increase in drug concentration, the concentrations of toxic ingredients also increased. This may have resulted in a decreased survival rate of RAW264.7 cells in the original inflammatory state. Second, the substance enters the cell through membrane dynamic transport and attaches to the cell with the help of certain specific proteins. Compared to the normal cell group, the intracellular drug concentration becomes higher and the toxicity increases, leading to a decrease in affinity and intracellular AUC. Finally, membrane permeability at high concentrations is limited, and when cells are damaged in the inflammatory state, some functional transporter proteins and drug metabolism enzymes (DMEs) on the surface of the cell membrane are altered accordingly, which causes a decrease in the intracellular accumulation of drugs.

When the administered dose and drug concentration exceed a certain limit, the enzyme catalytic capacity and carrier transport capacity are saturated, and its pharmacokinetics show a clear dose dependence. Nonlinear pharmacokinetic processes are subject to metabolic enzyme saturation, where enzyme metabolism is saturated, which will cause drug accumulation with an exponential increase in AUC, C_max_, and T_1/2_ (Ye, et al., 2020). In the body, the metabolic enzymes of some drugs have a limited capacity to metabolize them, and the administration of larger doses leads to a saturable metabolic process of the corresponding substrate drug. The blood concentration and the dose are not proportional at this time, which also leads to a significant increase in the body’s drug concentration and causes clinical effects and toxic side effects (Liu, 2016).

Moreover, carrier protein saturation has an effect on pharmacokinetic processes. Drug transport is influenced by transport proteins in the cell membrane, which can be divided into uptake proteins and efflux proteins. Nonlinear pharmacokinetics are regulated by uptake proteins and efflux proteins, resulting in nonlinear phenomena in the *in vivo* dynamics of the drug. As a result of saturation of absorbed proteins, the C_max_ or AUC does not increase proportionally with increasing doses, while the saturation of exocytosis proteins causes a decrease in exocytosis of secreted drugs and an increase in the AUC value with increasing concentrations. In the present research, the nonlinear kinetics are induced by the saturation of exocytosis proteins through AUC values/doses. In addition, drug efflux proteins, such as P-glycoprotein, are widely distributed on the cell surface. They are able to regulate the permeability of cell membranes and the intracellular concentrations of certain drugs. The involvement of efflux proteins may further decrease the intracellular drug concentration, and drug redistribution may also occur in subcellular organelles after cellular uptake (Zhang, et al., 2018; Liu, et al., 2019; Zhou, et al., 2020).

In addition, enzyme saturation has an effect on pharmacokinetic processes. There are two forms of intracellular elimination of drugs: cellular elimination and conversion by enzymatic regulation. The metabolism of most drugs is associated with cytochrome P450 (CYP) enzymes. The metabolic capacity of CYP enzymes is complex, and the profile of DMEs may change in pathological states, which may affect drug metabolism, as well as the body state, which may also affect drug distribution and elimination. It was found that macrophages attacked by LPS secrete large amounts of the inflammatory factor NO. Excessive NO reduces the affinity of cytochrome *c* for oxygen, which inhibits mitochondrial respiratory chain function, and NO combines with peroxides to form peroxynitrite anion (ONOO^−^), which can affect the function of glycolytic and respiratory enzymes and interfere with cellular energy metabolism (Ma et al., 2019). Clinical studies have shown that in some pathological states, such as inflammation, DME expression and activity can be reduced, resulting in a decrease of 20–70% in drug clearance (Bissery et al., 1996), which may also be responsible for the large individual differences in pharmacokinetic parameters of patients. Current reports suggest that acute inflammation may alter the pharmacokinetics of non-steroidal anti-inflammatory analgesics (Projean, et al., 2005; Uno, et al., 2008) and that such differences may arise from inflammation and fluctuations in CYP enzyme activity during drug administration (Gong et al., 2014). Our previous study found that *I. cappa* may inhibit this enzyme activity when combined with drugs metabolized via CYP3A, CYP2C9, CYP2C19, and CYP1A2 (Song et al., 2019). It is speculated that the five components may inhibit the activity of their own DMEs, thus causing slowed metabolism, decreased clearance, a prolonged half-life, and increased blood concentrations and AUC values. In the present study, five components showed a decrease in clearance with increasing concentrations. There was an increase or decrease in normal and inflammatory cells, suggesting that the expression of metabolic enzymes in cells may be different between components of *I. cappa* in the inflammatory state.

Changes in pharmacokinetic behavior and pharmacokinetic parameters are not determined by a single factor but may be the complex result of multiple factors. Cell permeability, cell surface transporters, and saturation of DMEs are altered in the inflammatory state, and these factors are key determinants of drug entry into target cells, intracellular metabolism, and the corresponding drug efficacy (Zhou, et al., 2011; Patrick, et al., 2016). The specific mechanisms underlying the differences in the pharmacokinetic processes of the five anti-inflammatory active ingredients in *I. cappa* are subject to further study.

## Data Availability

The original contributions presented in the study are included in the article/supplementary material, further inquiries can be directed to the corresponding authors.

## Abbreviations

LUT, Luteolin; CA, chlorogenic acid; CCA, cryptochlorogenic acid; 3,4-DCQA, 3,4-dicaffeoylquinic acid; 4,5-DCQA, 4,5-dicaffeoylquinic acid; LPS, lipopolysaccharide; NO, nitric oxide; TNF-α, tumor necrosis factor-α; CYP, cytochrome P450; DMEs, drug-metabolizing enzymes; T_1/2_ , terminal elimination half-life; AUC_0–t_, area under the concentration vs. time curve from zero to last sampling time; V_d_/F , volume of distribution; CL/F, total body clearance; C_max_, the peak concentration; T_max_, the time to reach C_max_.
